# Bone metastasis in differentiated thyroid cancer: Spanish multicenter study of clinical characteristics, survival and prognostic factors

**DOI:** 10.3389/fendo.2024.1466245

**Published:** 2024-11-07

**Authors:** Suset Dueñas-Disotuar, Ana Piñar-Gutiérrez, Irene de Lara-Rodríguez, Julia Sastre-Marcos, Emma Anda-Apiñániz, Amelia Oleaga-Alday, JC Galofré, Aida Orois, Victoria Alcázar-Lázaro, Laia Martínez-Guasch, Cecilia Sánchez-Ragnarsson, María Ángeles Gálvez-Moreno, Cristina Familiar-Casado, Tomás Martín-Hernández, Ana R. Romero-Lluch

**Affiliations:** ^1^ Department of Endocrinology, Hospital Universitario Virgen del Rocío, Sevilla, Spain; ^2^ Department of Endocrinology, Hospital Universitario de Toledo, Toledo, Spain; ^3^ Department of Endocrinology, Hospital Universitario de Navarra, Pamplona, Spain; ^4^ Department of Endocrinology, Hospital Universitario de Basurto, Universidad del País Vasco (UPV/EHU), Bilbao, Spain; ^5^ Department of Endocrinology, Clínica Universidad de Navarra, Pamplona, Spain; ^6^ Department of Endocrinology and Nutrition, Hospital Clínic, Barcelona, Spain; ^7^ Department of Endocrinology, Hospital Severo Ochoa, Leganés, Spain; ^8^ Department of Endocrinology, Hospital Joan XXIII, Tarragona, Spain; ^9^ Department of Endocrinology, Hospital Universitario Central de Asturias, Instituto de Investigación Sanitaria del Principado de Asturias, Oviedo, Spain; ^10^ Department of Endocrinology, Hospital Universitario Reina Sofía, Córdoba, Spain; ^11^ Department of Endocrinology, Hospital Clínico San Carlos, Madrid, Spain; ^12^ Department of Endocrinology, Hospital Universitario Virgen Macarena, Sevilla, Spain

**Keywords:** thyroid cancer, bone metastases, survival, skeletal-related events, radioiodine, multikinase inhibitors; antiresorptive agents

## Abstract

**Objective:**

This study describes the characteristics, survival and prognostic factors in a cohort of patients with bone metastases (BM) from differentiated thyroid carcinoma (DTC).

**Methods:**

This was a multicenter retrospective observational study conducted in Spain, including patients diagnosed with DTC and BM between 1980 and 2022. A Cox regression analysis was performed to examine prognostic factors for survival. Kaplan-Meier and log-rank tests were performed for survival analysis and comparison between groups.

**Results:**

A total of 133 patients were included with a median follow-up of 40 (17-70) months. Seventy patients (52.6%) had BM at the initial diagnosis. Fifty-two (39.1%) had follicular carcinoma. Sixty-six (49.6%) presented multiple BM. The most frequent location was the spine (63.2%). Other metastases were present at diagnosis in 88 (66.2%), mainly lung (60.9%). BM were treated with I131 in 91 (68.4%) patients, with BM uptake in 63 (47.4%). Fifty-six (42.1%) received treatment with multikinase inhibitors. Fifty-three (3.9%) had skeletal-related events. Seventy-two (54.1%) died. The 3-, 5- and 10-year survival was 53.5, 39.5% and 28.5%, respectively. Significant prognostic factors in the multivariate analysis were the presence of lymph node metastases (N1) HR 1.71 (95% CI 1.005-2.098; p=0.048), BM treatment with I131 HR 0.532 (95% CI 0.304-0.931; p=0.027) and age ≥67 years at BM diagnosis HR 1.991 (95% CI 1.142-3.47; p=0.015).

**Conclusions:**

Survival of DTC patients with BM treated in a Spanish cohort was 39.5% at 5 years and 28.5% at 10 years. Patients with BM treated with I131 appear to have a better outcome in terms of mortality and the presence of lymph node involvement and age over 67 years were associated with higher mortality.

## 1 Introduction

Differentiated thyroid carcinoma (DTC) is a neoplasm with a 5-year survival of over 98%. Bone metastases (BM) are present in 2-13% of patients (1) and are more common in follicular carcinomas (7-28%) (2). The prognosis worsens in patients who present BM, with survivals described in observational studies of 42-61% and 20-27% at 5 and 10 years, respectively (3–5). It has been shown that there are certain poor prognostic factors in this type of patient such as male sex (6), older age (6, 7), greater extension of BM (7), multiple BM (8), late detection of BM (7), metastases in other locations (5, 9), skeletal-related events (SRE) (6) and/or the absence of I131 treatment or no uptake of I131 by the BM (5, 9, 10).

BM may be present at diagnosis of DTC or diagnosed during follow-up, developing silently but with very high levels of thyroglobulin and/or anti-thyroglobulin antibodies or by associated symptoms such as pain, fractures, spinal cord compression or malignant hypercalcemia (11). Treatment of BM can be aimed at modifying the disease and therefore influencing survival, preserving/restoring the anatomy or palliating the symptomatology depending on the profile and prognosis of each patient. Current research is primarily focused on evaluating which therapies can be disease modifying and extend patient survival (2). Nevertheless, the available studies have not demonstrated a clear improvement in survival, showing a reduced response and lack of adequate clinical control with the classic therapies used in metastatic DTC when bone metastases are involved (7, 12, 13). Among the available treatment options are I131, tyrosine kinase inhibitors (TKIs), targeted therapy based on tumor mutations, radiotherapy, percutaneous procedures, surgery and antiresorptive drugs to palliate or delay the appearance of SRE, although their recommendations are based on real-life descriptions of short series of patients and on information on other more common tumors in which BM also occur (1). Regarding TKIs, data on BM secondary to DTC are limited to the SELECT (14) and DECISION (15) studies, in which lenvatinib and sorafenib, respectively, were shown to be modestly effective treatment.

The problem is that although BM are rare, they have a major negative impact on survival and there is currently little evidence on their clinical course and management. Moreover, this evidence is based on observational studies with small samples and heterogeneous results. In 2023, we published the results of our series of patients from Andalusia (5). In summary, survival was poorer in patients who were not treated with I131, had lymph node metastases and/or had other distant metastases. In view of these results, the Thyroid Knowledge Area of ​​the Spanish Society of Endocrinology and Nutrition (TiroSEEN) promoted the expansion of the study to a national level, with the aim of extending the information available on the characteristics and survival of these patients, while seeking to identify prognostic factors in a Spanish cohort of patients with BM from DTC.

## 2 Methods

### 2.1 Study design and patients included

A retrospective observational multicenter study was performed. Adult patients with BM from DTC treated in 15 hospitals in Spain between 1980 and 2022 were included. The study was carried out with the collaboration of the Thyroid Knowledge Area of ​​the Spanish Society of Endocrinology and Nutrition (TiroSEEN).

### 2.2 Variables collected

An Excel database was created and sent to all the participants to be filled in with patient data in a standardized manner. Subsequently, these data were sent in anonymized form to those responsible for the study.

The variables collected were sex, age (at diagnosis of DTC and BM), diagnosis of BM (metachronous vs synchronous), type of thyroid surgery, histologic variant, extrathyroidal extension, vascular invasion in the case of papillary carcinoma, presence of BRAF^V600E^ and/or TERT mutations, thyroglobulin level at DTC and BM diagnosis, stage according to the 8th edition of TNM (16), characteristics of BM (whether they were single or multiple, number, location), presence of metastases in other locations at diagnosis, treatments performed (antiresorptives, corticosteroids, radioactive iodine (RAI) remnant ablation dose, BM-specific RAI treatment dose and BM RAI uptake, radiotherapy, surgery and TKIs), and adverse effects, presence of SRE (fractures, spinal cord compression, hypercalcemia of malignancy, need for surgery due to pain and need for radiotherapy due to pain) and death (including reason for death).

### 2.3 Statistical analysis

The descriptive analysis was performed by obtaining the median and the quartiles for quantitative variables (expressed as P50 (P25-P75)) and frequency for qualitative variables (expressed as n (%)). The X^2^ test was used for the comparison of proportions. For the study of risk factors for mortality, a univariate analysis was performed using Cox regression and subsequently a multivariate Wald analysis with the variables with p<0.1 in the univariate analysis. For this, the variable of age at diagnosis of BM was categorized as older or younger than 67 years since this was the median age of our cohort. The overall survival analysis was performed using the Kaplan-Meier method and the log-rank test was used to compare survival between groups. A p-value less than 0.05 was considered statistically significant. The statistical analysis was conducted using the Statistical Package for the Social Sciences (SPSS®) version 29 (IBM Corporation, New York, USA) and R (free software) for Windows and was performed by the statistical staff of the Foundation for the Management of Health Research in Seville.

## 3 Results

### 3.1 Demographic and clinical characteristics

The baseline characteristics of the patients are shown in **Table 1**. A sample of 133 patients was obtained, 76 (57.1%) of whom were women. The median age at diagnosis of DTC was 64 years (range: 53-71) and at diagnosis of BM was 67 years (range: 57-73). Seventy (52.6%) patients presented BM at DTC diagnosis (synchronous).

**Table 1:** Description of baseline characteristics.

Variable	Result
Sex (female)	76 (57.1%)
Age at diagnosis of DTC (years) Age at diagnosis of BM (years)	64 (53-71) 67 (57-73)
Follow-up time from diagnosis of BM (months)	40 (17-70)
Metachronous BM	63 (47.4%)
Time between diagnosis of DTC and diagnosis of metachronous BM (months)	56 (21-116)
Follicular carcinoma Papillary carcinoma *Aggressive variants* Poorly differentiated Hürthle cells Unknown	52 (39.1%) 45 (33.8%) *15 (25%)* 8 (6%) 11 (8.2%) 2 (1.5%)
Mutation *BRAF^V600E^* *TERT* *Unknown*	3 (8.8%) 5 (31.2%) 35 (26.3%)
Invasion (follicular DTC) *Minimally invasive without angioinvasion* *Minimally invasive with angioinvasion* *Widely invasive* *Unknown*	4 (7.6%) 16 (30.7%) 17 (32.7%) 15 (28.8%)
Vascular invasion (papillary DTC)	24 (47%)
Extrathyroid extension *No* *Microscopic* *Macroscopic* *Unknown*	55 (41.4%) *15 (11.3%)* *20 (15%)* *43 (32.3%)*
Thyroglobulin after surgery (ng/mL) Thyroglobulin at last revision	500 (65-2703) 709 (67-5000)
T *X* *1* *2* *3a* *3b* *4* *Unknown*	*5 (3.8%)* *21 (15.7%)* *35 (26.3%)* *46 (34.5%)* *3 (2.3%)* *13 (9.8%)* *10 (7.5%)*
N0 N1 Unknown	62 (46.6%) 54 (40.6%) 17 (12.8%)
Multiple BM at Diagnosis of BM	66 (49.6%)
Location of BM *Brain* *Spine* *Rib cage* *Sternum* *Extremities* *Pelvis*	*24 (18%)* *84 (63.2%)* *26 (19.5%)* *11 (8.3%)* *50 (30.6%)* *47 (35.3%)*
Metastases in other locations at initial diagnosis *Lung* *Brain* *Liver* *Others*	88 (66.2%) *81 (60.9%)* *6 (4.5%)* *4 (3%)* *12 (9%)*

With respect to anatomic pathology, 52 (39.1%) patients had follicular carcinoma. Concerning mutations, only 34 reports included information on whether the BRAF^V600E^ mutation was present or absent, while 16 reports included information on TERT mutations. In the patients with follicular carcinoma, 19 (32.7%) had a largely invasive tumor, while in those with papillary carcinoma, 24 (47%) had vascular invasion.

Fifty-one patients (73.3%) had stage T3 or T4 according to TNM. Fifty-four (40.6%) had lymph node metastases, although this information was unknown for 17 (12.8%) patients. Eighty-eight (66.2%) had metastases in other locations at initial diagnosis, the most frequent being lung metastasis, present in 81 (60.9%). The most frequent location of bone metastases was the spine in 84 (63.2%) patients.

### 3.2 Treatments and adverse events

The treatments administered are shown in **Table 2**. It should be noted that 120 (90.2%) patients received RAI ablation therapy, of whom 64 (53.3%) had bone uptake. In 91 (68.4%) patients, RAI was administered at a second time point for BM treatment, with 63 (47.4%) presenting RAI uptake. Patients who received RAI therapy at a second time point for BM showed no significant differences compared to those who were not treated in terms of lymph node involvement (p=0.099), T stage (P=0.23), multiple BM at initial diagnosis (p=0.925), lung metastases (p=0.269) or metachronous BM (p=0.927). There were however differences in age: 65 (57-52) vs 71 (57-78); p=0.039.

**Table 2:** Treatments used in BM secondary to DTC.

Variable	Result
Type of surgery *Total thyroidectomy* *Total thyroidectomy + central lymph node dissection* *Total thyroidectomy + central and lateral lymph node dissection* *Hemithyroidectomy* *Data not available*	44 (33.1%) 25 (18.8%) 25 (18.8%) 2 (1.5%) 37 (27.8%)
RAI therapy *Remnant ablation* *I131 activity (mCi)* *BM RAI uptake* *BM RAI Treatment* *Number of doses used* *I131 activity (mCi)* *BM RAI uptake*	*120 (90.2%)* *150 (100-200)* *64 (48.1%)* *91 (68.4%)* *2 (1-3)* *365 (200-552)* *63 (47.4%)*
Antiresorptive treatment *Zoledronic* *Denosumab* *Alendronic acid*	41 (30.8%) *26 (19.5%)* *11 (8.3%)* *3 (2.3%)*
Corticosteroid treatment	45 (33.8%)
Surgical treatment of BM	49 (36.8%)
Radiofrequency	5 (3.8%)
Cryoablation	1 (0.8%)
Embolization	7 (5.3%)
Radiotherapy	86 (64.7%)
TKI *Sorafenib* *Lenvatinib* *Axitinib*	56 (42.1%) 31 (23.3%) 24 (18.8%) 1 (0.8%)

Radiotherapy was a common treatment used in 86 (64.7%) patients. With respect to other treatments, 56 (42.1%) patients received TKI therapy (31 with sorafenib, 24 with lenvatinib and one with axitinib). Thirty (53.5%) patients had complications associated with TKI therapy, 22 (39,2%) of them grade 3-4, four (7%) had renal complications, eight (14. 2%) hypertension, three (5%) osteonecrosis of the jaw (of these, one had not received treatment with bisphosphonates, one patient had also received treatment with denosumab and another with intravenous zoledronic acid), nine (16%) liver complications and seven (12.5%) other types of complications. When evaluating the response to TKI, nine (16%) presented disease stability, four (7.1%) partial response, 0% complete response and 21 (37.5%) progression. It was not possible to collect the data from the medical records of the remaining 22 patients on TKI therapy.

Antiresorptive therapy was administered in 41 (31.8%) patients, with five presenting adverse events associated with these treatments. Specifically, one suffered osteonecrosis of the jaw, three hypocalcemia and one lost multiple teeth. The median time between diagnosis of BM and initiation of antiresorptives was 6.5 months, with a p75 of 24 months.

Finally, patients with multiple BM at diagnosis were as likely as those with a solitary BM to receive RAI (p=0.925), corticosteroids (p=0.116), antiresorptives (p=0.248), radiotherapy (p=0.43) and TKI (p=0.101). The only difference in terms of the types of treatments used in these two situations was the performance of surgery for metastasis in patients with a single vs multiple BM (62.5% vs 31.8%).

**Figure 1:**
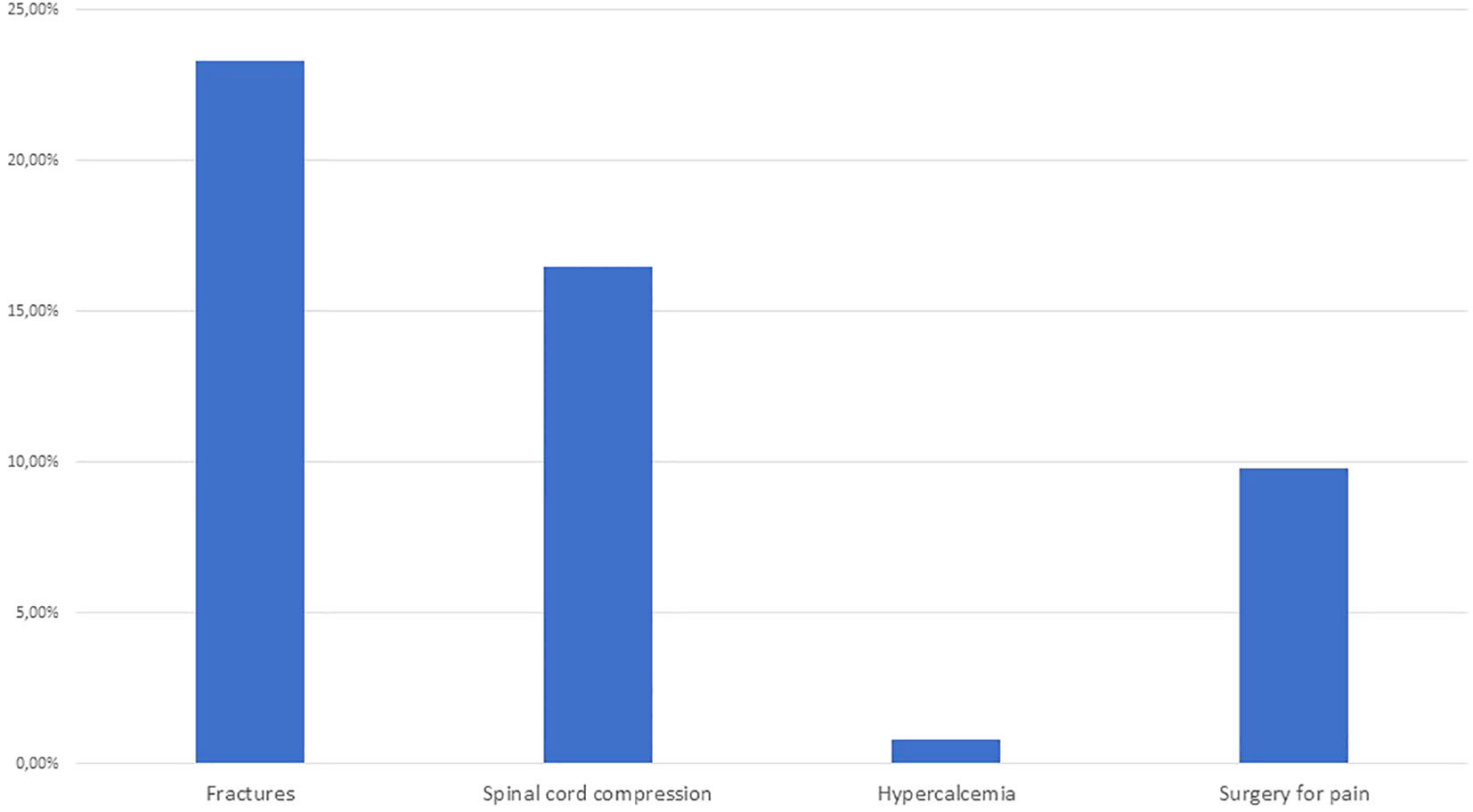
Frequency of SRE in patients with BM secondary to DTC.

Overall, 53 (39.8%) patients had SRE. These are shown in **Figure 1**.

### 3.3 Mortality

During follow-up, 72 (54.1%) patients died. Of those with a recorded cause of death (67 patients), 56(83.5%) died from causes associated with DTC and 11(16.5%) died from non-associated causes. The exact cause of death could not be established for the rest of the patients because of incomplete recording due to the age of the cases. The age at death was 73 (67-77) years. The time between DTC diagnosis and death was 5 (2-9) years. In patients with synchronous BM, the time between BM diagnosis and death was 2 (1-4) years, and in patients with metachronous BM, the time between BM diagnosis and death was 1 (0-3) years, although there was no statistically significant difference between the two groups (p=0.687).

### 3.4 Prognostic factors for mortality

In the univariate analysis, the factors associated with an increased risk of mortality were age ≥67 years, metachronous BM, the presence of lymph node (N1), pulmonary metastases and spinal cord compression. RAI therapy for BM and RAI uptake by BM were found to be protective factors. In the multivariate analysis, age ≥67 years (HR 1.991 (95% CI 1.142-3.47); p=0.015) and the presence of lymph node metastases (HR 1.71 (95% CI 1.005-2.098); p=0.048) were statistically significant as the worst prognostic factors, and RAI treatment of BM (HR 0.532 (95% CI 0.304-0.931); p=0.027) as a protective factor (**Table 3**).

**Table 3:** Results of the univariate analysis of risk factors for death.

	Univariate analysis		Multivariate analysis	
Variable	OR (CI 95%)	*P*	OR (CI 95%)	*P*
**Age** ≥67 years*	2.38 (1.41-4.01)	0.001	1.991 (1.142-3.47)	0.015
BM RAI therapy*	0.45 (0.27-0.75)	0.002	0.532 (0.304-0.931)	0.027
No BM RAI uptake*	2.91 (1.59-5.33)	<0.001		
**Multiple BM at diagnosis ^a^**	2.47 (0.88-6.92)	0.085		
Lymph node metastases*	2.08 (1.23-3.52)	0.006	1.71 (1.005-2.098)	0.048
Metachronous BM*	1.9 (1.15-3.12)	0.012		
Lung metastases*	3.29 (1.02-10.6)	0.046		
Spinal cord compression*	1.94 (1.08-3.49)	0.026		

*p<0.05 ^a^p<0.1.

### 3.5 Survival analysis

**Figure 2:**
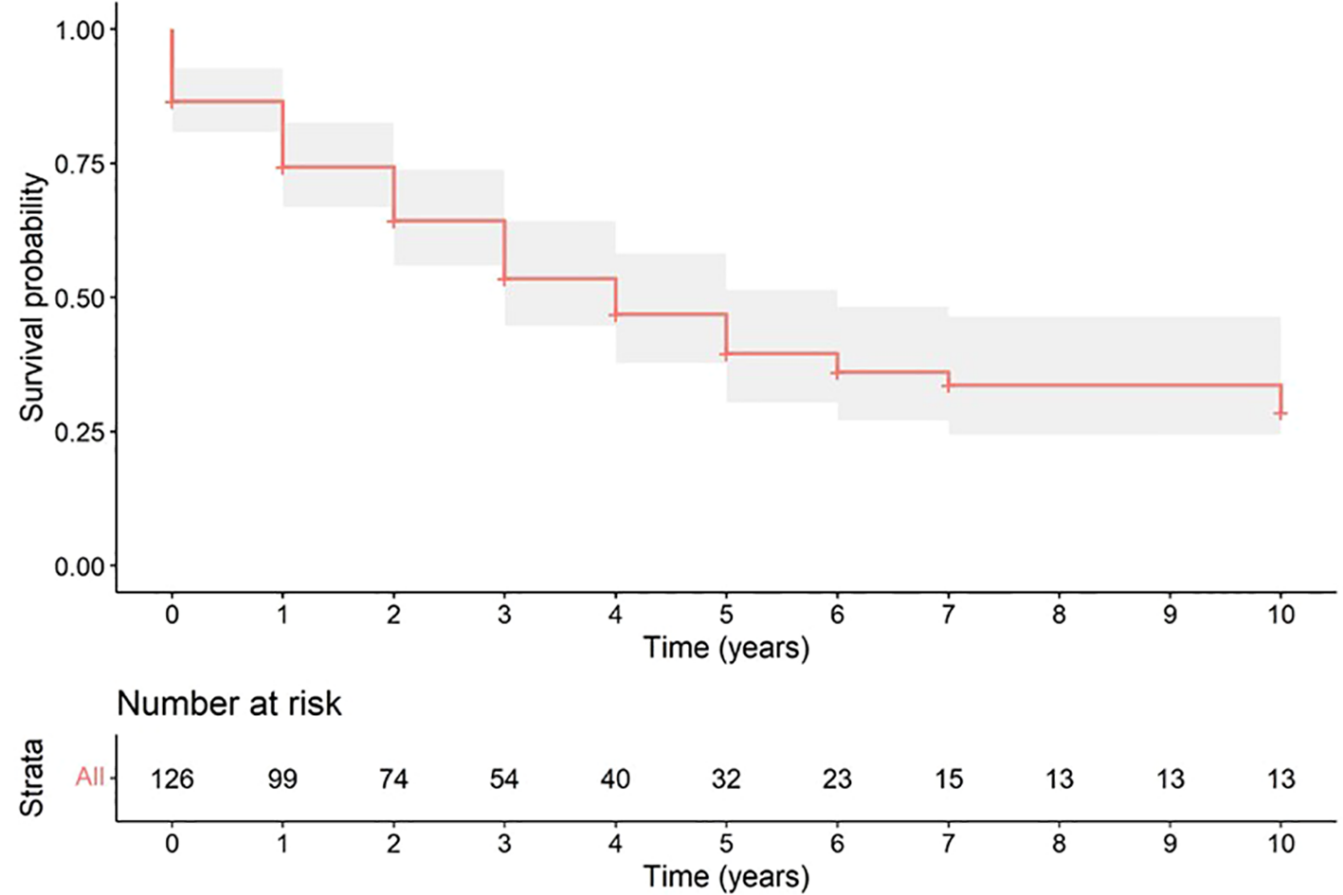
Plot of overall survival of patients with BM secondary to DTC.

Overall survival at 3 years after BM diagnosis was 53.5%, at 5 years 39.5% and at 10 years 28.5% (**Figure 2**).

**Figure 3:**
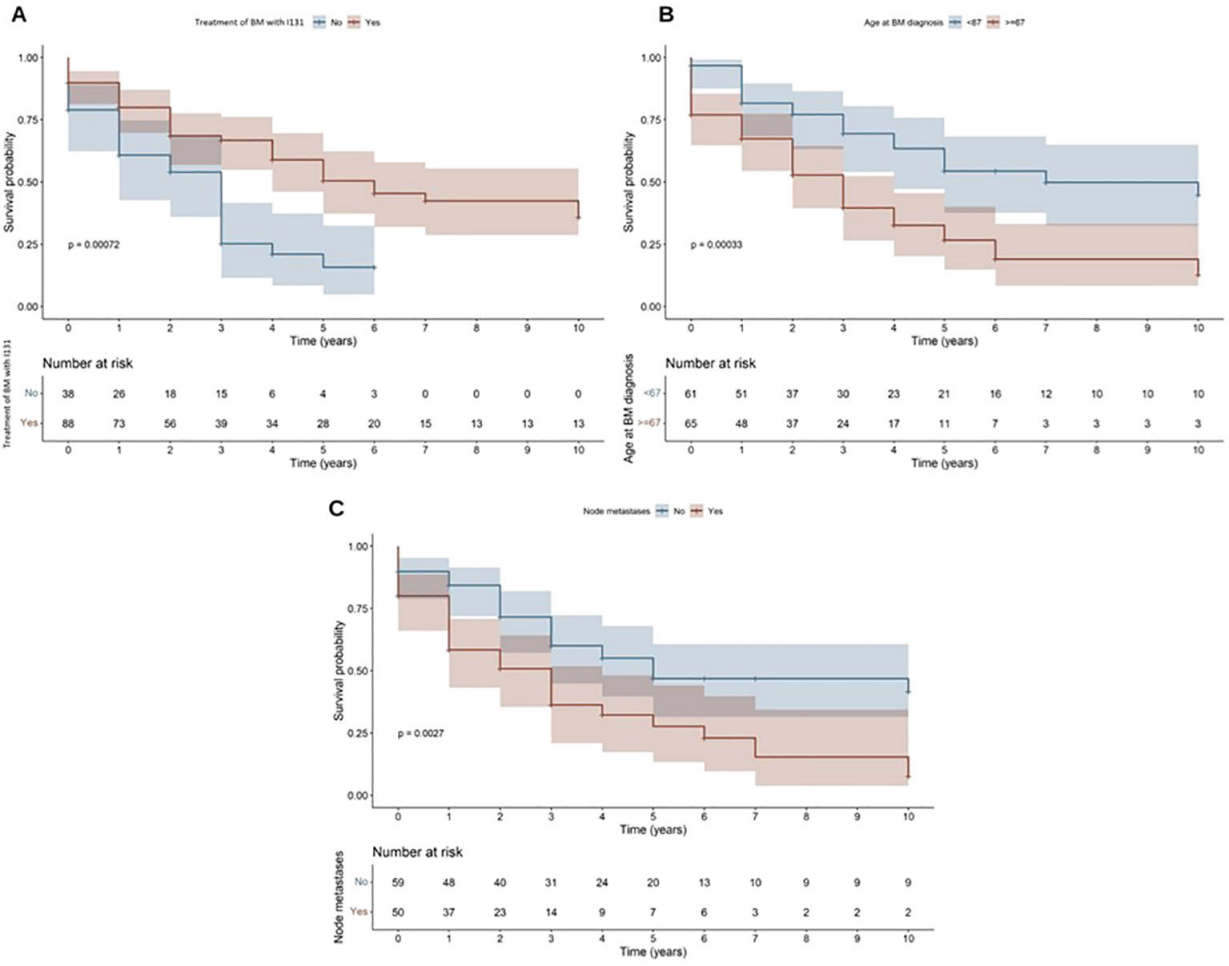
Survival plots according to: I131 therapy for BM **(A)**; Age at BM diagnosis (older or younger than 67 years) **(B)**; and presence of lymph node metastases **(C)**.

The survival plots according to the factors that reached statistical significance in the multivariate analysis are shown in **Figure 3**.

## 4 Discussion

The available studies on BM in patients with DTC are relatively few, and generally observational and retrospective in nature, including small cohorts of patients. The study with the largest number of patients was based on the US national database (Medicare) and included 1173 patients (5, 6). In August 2023 we published the results of a multicenter cohort from Andalusia, a region in southern Spain (8). In summary, we included 63 patients with BM from DTC, and the factors that were associated with poorer survival were lymph node involvement, presence of other metastases and not having been treated with RAI.

A number of other interesting studies have recently been published on BM from DTC. The first is that of Khired et al. (4), which used a population-based database of thyroid cancer patients from the United States and included 976 patients with BM, although this study also included patients with medullary and anaplastic thyroid carcinomas. The study sought to compare patients with and without BM to assess which factors lead to the appearance of BM and which worsen prognosis. The second study (17) uses the same database and includes 579 patients diagnosed between 2010 and 2016. This study aimed to use machine learning to create predictive models of worse prognosis. Finally, Kanankulam Velliangiri et al. (18) studied 347 patients with BM from DTC and evaluated the risks and benefits of treating these patients with high doses of RAI.

The purpose of this new study was to extend our previous research with a larger number of patients and with a greater diversity of care since in Spain each Autonomous Community has a different health system, although it is always based on the same public health care model. This study therefore included 133 patients treated in 15 different centers throughout Spain. The demographic characteristics were similar to those of the Andalusian cohort: the median age at diagnosis of BM was 67 years and 42.9% were male. The median follow-up in months was also similar (35 vs 40 months). Regarding DTC characteristics, 52.6% had BM at initial DTC evaluation, 50.4% had a single BM and 66.2% had metastases in other locations. The percentage of patients with follicular carcinoma was 39.1%, and the presence of lymph node involvement, a factor associated with lower survival in both studies, was also present in a very similar percentage in both groups (40.6% and 41.3%).

The age of the patients in our cohort was similar to that reported by Mazziotti et al. (10), but higher than that published by other authors such as Kanankulam Velliangiri et al. (18). With respect to distribution by sex, the percentage of men is generally lower than that of women, but this varies from 29% to 47.1% (4, 18). It should be noted that the percentage of patients with follicular carcinoma in our study (39.1%) was slightly higher than the percentage of patients with papillary carcinoma (38.3%), although it is known that in some series the percentage of papillary carcinoma may be higher than that of follicular carcinoma precisely because of its greater frequency. However, other studies have shown that follicular carcinoma was more frequent (18).

Regarding lymph node involvement, the percentages described vary from 29.7% to 45.6%, which may have an influence when evaluating prognostic factors and comparing them with our study (4, 10, 17). Concerning the extent of the disease at initial diagnosis, the data on lung metastases in our study are high in comparison with the others, with prevalences of 42-44.7% having been described in studies of large samples based on databases from the United States (4, 17) compared to our 60.9% in the Spanish cohort and 69.8% in the regional cohort. Finally, in our study, 47.4% of the BM were metachronous, a figure similar to that of Mazziotti et al. (10) (49.7%) but much higher than that of Kanankulam Velliangiri et al. (18) (22.4%).

The percentage of patients who received RAI treatment was also similar in the study by Mazziotti et al. (10) although their patients received a higher median total dose of I131 (563 vs 350 mCi). Antiresorptive therapy was used in only 30.8% of patients, showing that the use of these drugs is low and late considering the published role in the prevention of SRE in other tumors (1). SRE were less frequent (39.8%) than in our regional study (54%) and similar to that of Mazziotti et al. (10). The percentage of fractures was similar in the three studies, between 23.3% and 25.4%, but the percentage of spinal cord compression, 11.9-12.7% in the other two studies, was higher in this national cohort (16.5%). Again, these drugs were prescribed more as a treatment of SRE rather than to prevent them, which is something we should improve in our daily clinical practice, considering that only 12% of patients receiving them had related adverse effects. Finally, the percentage of patients on TKI treatment was similar to that of the Andalusian cohort (39.7% vs 30.8%), but lower than that reported by Khired et al. (4) (66.5% in patients with BM, although mention “systemic therapy” and not TKI specifically) and higher than the percentages reported by Kanankulam Velliangiri et al. (18) (11%) and Shi et al. (17) (17.4%).

Regarding mortality, the studies by Khired et al. (4) and Kanankulam Velliangiri et al. (18) presented similar percentages of death to ours (58.3% vs 52% vs 54.1%, respectively). This was approximately double that of the study by Mazziotti et al. (10), which may be because the cohort in this study had less lymph node involvement and lung metastases.

Overall survival at 5 and 10 years was 39.5% and 28.5%, respectively. In the study by Kanankulam Velliangiri et al. (18), the 5- and 10-year survival was 55.7% and 28.4%, respectively. The 5-year survival in the study by Khired et al. (4) was 41.7%. Survivals described in previous studies at 10 years vary from 13% to 50% (19, 20), which is due to methodological differences between studies. What is clear is that the appearance of these BM results in a significant decrease in survival in a tumor that by definition has a good prognosis, so much so that survival 5 years after diagnosis of DTC is >90% vs 39% in the case of BM.

In this study, the factors associated with lower survival were lymph node involvement and age ≥67 years. The factor associated with longer survival was the treatment of BM with RAI, using even lower total doses than in other studies. The association between RAI therapy and longer survival has been described in other studies (3, 9, 10, 17, 18). Other treatments failed to reach statistical significance when examined for their association with survival. However, this may be due to the small sample size of patients who were treated with the different possible modalities. Other recent studies have associated radiotherapy with (18) with increased survival.

The univariate analysis showed that the presence of simultaneous lung metastases could be associated with increased mortality, but it was not possible to demonstrate this in the multivariate analysis. In this national study, the very high prevalence of lung metastases (60.9%) resulted in this variable being poorly discriminative, and thus it had to be removed from the multivariate analysis model. Since other studies have been able to statistically demonstrate the worse prognosis of patients with lung metastases (8), this issue should be clarified in future studies.

Two other variables were significantly associated with poor prognosis in the univariate analysis: metachronous BM and multiple BM at initial diagnosis. Some studies have reported a relationship between the presence of multiple BM (8, 21–24) and metachronous BM (10) and a worse prognosis (8, 23, 24). Regarding multiple BM, it should be noted that this is not due to differences in the treatment used, but rather to a higher tumor disease burden and possibly also to the presence of metastases in other locations.

Prognostic variables described in other studies that we could not confirm are male sex (18), DTC size (17) metastases in other locations (5, 17), lung metastases (5), liver metastases (17), presence of SRE (6, 10, 25), location in the spine (26), BM surgery (8) and treatment with zoledronic acid (27).

The main limitations of the study are its observational and retrospective nature and its relatively small sample size despite the multicenter collaboration, which makes it difficult to obtain statistically significant results that allow us to establish clear and clinically applicable prognostic factors, as well as to determine which treatments may be more effective in such a complex and heterogeneous scenario as is the management of BM. There were also variables for which we had had little data because of their scarce use in previous decades, which made it difficult to obtain statistical significance. An important example would be mutations in BRAF^V600E^ and TERT. Their inclusion henceforth in studies of surgical specimens with DTC will be important in the future to evaluate their implication in the prognosis and targeted treatment of these patients. Finally, the lack of a joint protocol for the care of these patients in the different centers studied could also imply a bias when interpreting some results, since different treatments could have been applied to different patient profiles, but this also enriches the study if we wish to offer a real vision of the current care of these patients in Spain. The strengths of our study are the inclusion of a larger number of patients and its multicentricity, as well as the long follow-up time included.

## 5 Conclusion

This study presents data on the management and results in real clinical practice of patients with BM from DTC treated in different centers in Spain. Overall survival at 5 and 10 years was 39.5% and 28.5%, respectively. These findings are consistent with the current literature. Treatment of BM with RAI was a factor associated with better survival, while the presence of lymph node involvement and age greater than or equal to 67 years were associated with a worse prognosis. The development of national or multinational databases of patients with BM from DTC and the publication of their results would contribute to a better understanding of this disease and the treatments that can improve its prognosis.

## Data Availability

Requests to access the datasets should be directed to anapinarg@gmail.com.
