# Corrigendum: Bone metastasis in differentiated thyroid cancer: Spanish multicenter study of clinical characteristics, survival and prognostic factors

**DOI:** 10.3389/fendo.2024.1542771

**Published:** 2025-01-08

**Authors:** Suset Dueñas-Disotuar, Ana Piñar-Gutiérrez, Irene de Lara-Rodríguez, Julia Sastre-Marcos, Emma Anda-Apiñániz, Amelia Oleaga-Alday, JC Galofré, Aida Orois, Victoria Alcázar-Lázaro, Laia Martínez-Guasch, Cecilia Sánchez-Ragnarsson, María Ángeles Gálvez-Moreno, Cristina Familiar-Casado, Tomás Martín-Hernández, Ana R. Romero-Lluch

**Affiliations:** ^1^ Department of Endocrinology, Hospital Universitario Virgen del Rocío, Sevilla, Spain; ^2^ Department of Endocrinology, Hospital Universitario de Toledo, Toledo, Spain; ^3^ Department of Endocrinology, Hospital Universitario de Navarra, Pamplona, Spain; ^4^ Department of Endocrinology, Hospital Universitario de Basurto, Universidad del País Vasco (UPV/EHU), Bilbao, Spain; ^5^ Department of Endocrinology, Clínica Universidad de Navarra, Pamplona, Spain; ^6^ Department of Endocrinology and Nutrition, Hospital Clínic, Barcelona, Spain; ^7^ Department of Endocrinology, Hospital Severo Ochoa, Leganés, Spain; ^8^ Department of Endocrinology, Hospital Joan XXIII, Tarragona, Spain; ^9^ Department of Endocrinology, Hospital Universitario Central de Asturias, Instituto de Investigación Sanitaria del Principado de Asturias, Oviedo, Spain; ^10^ Department of Endocrinology, Hospital Universitario Reina Sofía, Córdoba, Spain; ^11^ Department of Endocrinology, Hospital Clínico San Carlos, Madrid, Spain; ^12^ Department of Endocrinology, Hospital Universitario Virgen Macarena, Sevilla, Spain

**Keywords:** thyroid cancer, bone metastases, survival, skeletal-related events, radioiodine, multikinase inhibitors, antiresorptive agents

In the published article, two author names were incorrectly written as “Juan Carlos Galofré-Ferrater” and “Aida Orois-Añón”. The correct spelling for these author names is “JC Galofré” and “Aida Orois”.

The authors apologize for this error and state that this does not change the scientific conclusions of the article in any way. The original article has been updated.

